# The Role of Notch and Wnt Signaling in MSC Communication in Normal and Leukemic Bone Marrow Niche

**DOI:** 10.3389/fcell.2020.599276

**Published:** 2021-01-08

**Authors:** Paul Takam Kamga, Riccardo Bazzoni, Giada Dal Collo, Adriana Cassaro, Ilaria Tanasi, Anna Russignan, Cristina Tecchio, Mauro Krampera

**Affiliations:** ^1^Stem Cell Research Laboratory, Section of Hematology, Department of Medicine, University of Verona, Verona, Italy; ^2^EA4340-BCOH, Biomarker in Cancerology and Onco-Haematology, UVSQ, Université Paris Saclay, Boulogne-Billancourt, France; ^3^Department of Immunology, Erasmus University Medical Center, Rotterdam, Netherlands; ^4^Hematology Unit, Department of Oncology, Niguarda Hospital, Milan, Italy; ^5^Department of Health Sciences, University of Milan, Milan, Italy

**Keywords:** Mesenchymal stromal cells, Notch, Wnt, leukemia, bone marrow niche

## Abstract

Notch and Wnt signaling are highly conserved intercellular communication pathways involved in developmental processes, such as hematopoiesis. Even though data from literature support a role for these two pathways in both physiological hematopoiesis and leukemia, there are still many controversies concerning the nature of their contribution. Early studies, strengthened by findings from T-cell acute lymphoblastic leukemia (T-ALL), have focused their investigation on the mutations in genes encoding for components of the pathways, with limited results except for B-cell chronic lymphocytic leukemia (CLL); in because in other leukemia the two pathways could be hyper-expressed without genetic abnormalities. As normal and malignant hematopoiesis require close and complex interactions between hematopoietic cells and specialized bone marrow (BM) niche cells, recent studies have focused on the role of Notch and Wnt signaling in the context of normal crosstalk between hematopoietic/leukemia cells and stromal components. Amongst the latter, mesenchymal stromal/stem cells (MSCs) play a pivotal role as multipotent non-hematopoietic cells capable of giving rise to most of the BM niche stromal cells, including fibroblasts, adipocytes, and osteocytes. Indeed, MSCs express and secrete a broad pattern of bioactive molecules, including Notch and Wnt molecules, that support all the phases of the hematopoiesis, including self-renewal, proliferation and differentiation. Herein, we provide an overview on recent advances on the contribution of MSC-derived Notch and Wnt signaling to hematopoiesis and leukemia development.

## Introduction

Bone marrow microenvironment (BMME) supports normal and clonal hematopoiesis, but also affects leukemia initiation, progression, and chemoresistance. Hematopoietic stem cells (HSCs) reside in a specialized BMME, where HSCs are tightly regulated (Cordeiro-Spinetti et al., 2015), functionally subdivided in two main compartments, i.e., the vascular niche that is close to the marrow vasculature, and the endosteal niche that is close to endosteum; however, the specific nature and functions of each niche still remain unclear (Morrison and Scadden, 2014; Calvi, 2020). Within BM niches, HSCs interact with cellular components, including endothelial cells (ECs), mesenchymal stromal cells (MSCs), megakaryocytes (MKs), osteoblasts (OBs), specialized macrophages, and nerve fibers (Calvi et al., 2003; Wilson et al., 2007). The redundant and complex activity shared by these cellular components has made difficult the assessment of the precise role of each cell type. However, these cells are dynamically involved in the regulation of hematopoiesis, through soluble or membrane-bound molecules (receptors and ligands) (Morrison and Scadden, 2014). MSCs include adult stem cells with multilineage differentiation capacity, that give rise to many other stromal cell types, including osteoblasts, adipocytes, chondrocytes, and endothelial cells (Dominici et al., 2006). As observed, both *in vitro* and in animal models, MSCs are capable of reconstituting a functional hematopoietic microenvironment, expressing/producing cytokines, and growth factors necessary for the regulation of hematopoiesis (Muguruma et al., 2006; Pontikoglou et al., 2011). Consequently, MSCs are largely used in 2D and 3D *in vitro* or *ex vivo* co-culture systems as a surrogate of the BMME, thus representing a suitable model for evaluating the role of BMME on HSCs and leukemic cells (Jakubikova et al., 2016). MSCs, by either producing cytokines and chemokines or entering in direct contact with leukemia cells, activate cell transduction signals that tightly regulate normal and malignant hematopoietic cell survival, thus driving the chemoresistance-promoting effect of the BMME (Jacamo et al., 2014). Our and other groups have demonstrated that Notch and Wnt signaling pathways represent a major crosstalk used by MSCs to interact with BMME (Kamdje et al., 2011, 2012; Zhang et al., 2013; Takam Kamga et al., 2016a). Indeed, these two pathways are well documented for their pivotal functions during normal and malignant hematopoiesis. Even though their deep role is well known in some leukemia subtypes, such as T-ALL, they can play opposite functions, being either oncogenic or tumor suppressor. However, all studies eventually unravel a conserved and supportive role for MSC-derived Notch and Wnt pathways in leukemia.

## MSCs

Mesenchymal stem/stromal cells (MSCs) are multipotent non-hematopoietic cells with multilineage differentiation capacity. According to ISCT (International Society for Cellular and Gene Therapy, MSCs could be defined according to three criteria; (i) spindle shaped and plastic-adherent cells in standard tissue culture plates; (ii) expression of mesenchymal markers (CD105+, CD73+, CD90+) and lack of hematopoietic markers (CD45-, CD34, CD14- or CD11b-, CD79a or CD19, and HLA-DR), and (iii) *in vitro* multipotent capability of differentiating into osteocytes, adipocytes, and chondrocytes (Dominici et al., 2006). There are several sources of MSCs including BM, cord blood, adipose tissue, and others (Krampera et al., 2007; Di Trapani et al., 2013; Petrenko et al., 2020). MSCs have become widely studied over the past 30 years for their potential clinical application in tissue engineering and regenerative medicine for bone and cartilage reconstruction and wound healing. Actually, *in vitro* and *in vivo* data support the evidence that one of the most important biological properties of MSCs is the immunoregulatory effect toward innate and adaptive immune effectors cells, such as T-, B-, and NK-cells, monocytes and dendritic cells in different inflammatory conditions, such as graft-versus-host disease (GvHD) (Collo et al., 2020). Indeed, migration, secretion, tissue regeneration, and immune regulatory properties of MSCs are synergistic and frequently rely on common signaling pathways, such as bone morphogenetic proteins (BMP) (Kong et al., 2013), platelet-derived growth factor (PDGF), Wnt, and Notch, especially inside BMME. Leukemia cells can interfere with the modulation of these pathways to improve biological function of MSCs toward a pro-leukemia supportive effect (Wang et al., 2015, 2016).

## Notch Signaling in MSCs

### Notch Signaling: Structure and Activation

**FIGURE 1:**
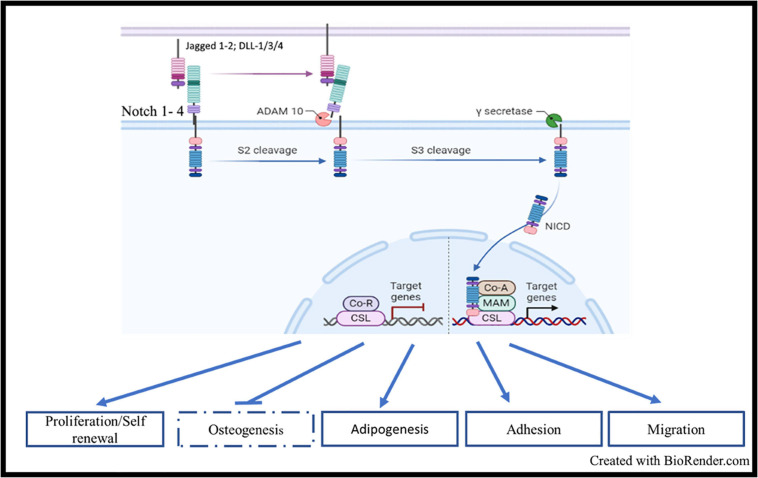
Notch signaling, structure, and activation: Mammal Notch system involved 4 receptors (Notch 1, Notch 2, Notch 3, and Notch 4) and 5 ligands of the Delta/Serrate/lag-2 (DSL) [Delta-like ligands (DLL-1, 3-4), Jagged1, and Jagged2]. Interaction between ligand and receptor expressed on adjacent cells induces two proteolytic events S2 and S3, catalyzed by ADAM-like metalloprotease and gamma-secretase complex, respectively. These two proteolytic events lead to the release of the intracellular active form of the receptor, i.e., NICD. NICD enters into the nucleus and forms a transactivation complex in association with partners, such as Master-mind like-1 (MALM1), Recombining binding protein suppressor of hairless/Core Binding Factor-1, Suppressor of Hairless, Lag-2 (RBP-jk/CSL). This transcription complex promotes the expression of genes of the helix basic family, including *HES1*, *HEY1*, and many other genes such as *NF-κB*, *MYC* and *CCNDD*.

Notch signaling is a master and evolutionary pathway conserved from flies to human (Ntziachristos et al., 2014). The term Notch is related to the notched wing phenotype observed in flies carrying notch gene haploinsufficiency, as Notch is involved in tissue patterning (Morgan, 1917). Mammal Notch system involved 4 receptors of Lin/Notch family (Notch 1, Notch 2, Notch 3, and Notch 4) and 5 ligands of the Delta/Serrate/lag-2 (DSL) [Delta-like ligands (DLL-1, 3-4), Jagged1 and Jagged2] (Figure 1; Gordon et al., 2008; Ables et al., 2011). Notch receptors are single-pass transmembrane receptors, containing three domains: an extracellular domain, a transmembrane domain and an intracellular domain, the latter known as Notch intracellular domain (NICD). The extracellular unit consists of an epidermal growth factor (EGF)-like repeat domain, which participates to the ligand binding. There are 36 EGF-repeats domains in Notch1 and Notch 2, and 34 and 29 repeats for Notch3 and Notch 4, respectively. EGF-like repeats are followed by a Lin12/Notch/repeats (LNR) structure acting as a negative regulatory region (NNR), by preventing the ligand-independent cleavage of the receptor. The NICD presents the RBP-J-associated molecule (RAM) domain, six ankyrin repeats (ANK), nuclear localization sequences (NLS), a transactivation domain (TAD) required for activating transcription, and a proline-, glutamate-, serine-, and threonine-rich (PEST) domain which regulates NOTCH degradation. Initially, Notch receptors are transcribed and translated as 210–300 kDa large precursor molecules. A series of post-translational modifications are required for the precursors to acquire their active form. The intact precursor molecules are first glycosylated in the endoplasmic reticulum (ER) by O-fucosyletransferase (Pofut-1 in mammals), which adds fucose to serine or threonine sites on specific EGF-like repeats. The glycosylated precursors are then cleaved in the trans-Golgi network into two subunits by furin-like convertases (S1-cleavage). This cleavage converts the precursor molecule into the non-covalently linked Notch extracellular domain (NECD) and transmembrane-Notch intracellular domain (TM–NICD) complex. This is then further glycosylated by enzymes of the Fringe family and addressed at cell membrane, where it is then non-covalently associated as a single heterodimer, i.e., the Cterm corresponding to the PEST domain and the Nterm corresponding to the extracellular region. Interaction between ligand and receptor expressed on adjacent cells induces two proteolytic events S2 and S3, catalyzed by ADAM-like metalloprotease and gamma-secretase complex, respectively. These two proteolytic events lead to the release of the intracellular active form of the receptor, i.e., NICD (van Tetering and Vooijs, 2011). NICD enters into the nucleus and forms a transactivation complex in association with partners, such as Master-mind like-1 (MALM1), Recombining binding protein suppressor of hairless/Core Binding Factor-1, Suppressor of Hairless, Lag-2 (RBP-jk/CSL). This transcription complex promotes the expression of genes of the helix basic family, including Hes1, Hey1, and many other genes, such as NF-κB, Myc, and cyclin D, thus controlling cell proliferation, apoptosis, adhesion, invasion, and migration during development, organ patterning and developmental diseases (Figure 1; Gordon et al., 2008).

### Notch Signaling in MSCs

As stemness signaling mediators, Notch components are expected to be present in MSCs (Moriyama et al., 2018). A comprehensive review of the literature reveals the presence of the transcript of all the four Notch receptors and ligands in MSCs (Zhang et al., 2019). Protein analysis through western immunobloting and flow cytometry supports the membrane expression of the four receptors. Western blot analysis showed that the proteins can be expressed as full length (220–280 kDa) transmembrane domains (90–110 kDa) (Takam Kamga et al., 2016a). Concerning ligands, most studies addressed the presence of Jagged1, while the expression of the other ligands are study-dependent. In general, DLL1, DLL-4, and Jagged 2 in less extend are reported, while a few studies support the expression of DLL-3. We observed that the expression of Notch ligands become readily detectable after 3 days of MSC culture (Kamdje et al., 2011), supporting the critical contribution of the physiologic state of MSCs when they are analyzed for Notch. In addition, MSCs in culture lose their stem cell-like properties after several subsequent passages; as Notch expression is negatively related to MSC senescence, cell passage should be considered when analyzing Notch expression (Mutyaba et al., 2014). Overall, MSCs express both Notch receptors and ligands, supporting the autocrine activation of Notch signaling. Nevertheless, mRNAs but not the related proteins of Notch target genes of the helix basic family, including Hes1, Hey, and He5 are represented in MSCs (Song et al., 2015). This observation is strengthened by the absence of cleaved form of Notch receptors in MSCs from healthy donors. Accordingly, MSC viability and differentiation are not affected by Notch pharmacological inhibitors, except for higher dose. It is unclear why the pathway is not active, regardless the presence of receptors, and ligands, but it is possibly due to postrancriptional repression mechanisms. Lessons from developmental biology may shed some light. During tissue development, Notch signaling on adjacent cells is involved in a phenomenon of *trans*/*cis*-activation/inhibition called lateral inhibition/activation. This model supports the idea that during tissue specification, the activation/inhibition of the signaling occurs among adjacent cells with opposite fate, while the involvement of the pathway is poor among similar cells (Sato and Yasugi, 2020). Notch signaling is activated either as paracrine signal to mediate communication between two different cell types or as molecular event involving stem cells differentiation. The first involvement will be discussed in another section. Concerning Notch involvement in stem cell differentiation, osteoblast switch is the paradigm. Cao et al. observed that the Notch inhibitor DAPT or a specific Notch1 antagonist may reduce alkaline phosphatase (ALP) activity in MSCs undergoing BMP9-dependent osteoblast induction, thus leading to reduced osteogenic differentiation *in vitro* and *in vivo*. On the other hand, MSC treatment with DLL-1 enhances ALP, osteopontin (OPN) and osteocalcin (OCN) expression (Cao et al., 2017). Using lentiviral tools, Semenova et al. (2020) proposed that Notch-promoting osteogenesis is dose-dependent, because the pathway activation is required for the formation of osteoblasts, but higher activity of Notch leads to apoptosis. The involvement of Notch for osteoblast differentiation has been confirmed by many other studies. Cao et al. (2017) has stressed the specific involvement of Notch1 and DLL-1, but other receptors or ligands could participate to Notch activation during osteogenesis. Song et al. (2015) observed that adipocyte differentiation is associated with reduced expression of Notch signaling components, suggesting that Notch involvement during MSC differentiation is lineage-dependent, i.e., down-regulated for adipogenic differentiation and activated for osteogenic differentiation. This could be related to the tight crosstalk between Notch and BMP/Smad/runx2 signaling. Similarly, the involvement of Notch signaling in other MSC properties are mainly related to the crosstalk with specific signals. For example, through the stabilization of hypoxia-inducible factor 1 alpha (HIF-1α), hypoxia improves several MSC functions, including cell adhesion, migration, and proliferation. Ciria et al. (2017) observed that hypoxia upregulates the expression and activation of Notch signaling, while the absence of Notch signaling impairs HIF1α-induced MSC adhesion, migration, and proliferation. Lessons from hypoxia models have been very useful to understand that Notch can modulate almost all the MSC functions. Considering that Notch signaling is required for all these hypoxia-mediated events, we can therefore propose a model where the pathway itself is a pivotal signal required for all MSC features.

## Wnt Signaling in MSCs

### Wnt Signaling Structure and Activation

**FIGURE 2:**
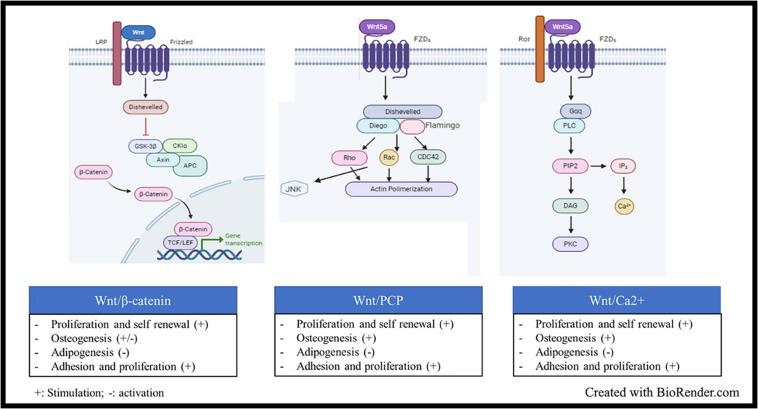
Wnt signaling, structure and activation: Wnt signaling is activated when glycoprotein ligands of the Wnt family bind Frizzled transmembrane receptors located at cell membrane level. Upon ligand binding, several cascades could be activated: (i) The Wnt/β-catenin, the ligand binds to the frizzled receptors and its coreceptors LRP5/6, which recruits the Disheveled (Dvl) protein, which in turn binds to Axin and GSK-3 proteins, leading to the disassembling of the β-catenin destruction complex, the subsequent release of β-catenin and its nuclear localization In the nucleus, β-catenin interacts with LEF/TCF transcription factors and other transcriptional activators to trigger activation of Wnt target genes. (ii) The Wnt planar cell polarity (PCP) pathway, the ligand binding on Frizzled receptors shows the recruitment of Dvl, the scaffold proteins Diego and Flamingo and the formation of a protein platform triggering the activity of Rho family GTPase proteins to regulate actin organization and cytoskeleton dynamics. (iii) the Wnt/Ca^2+^ which controls the levels of intracellular Ca^2+^. Upon ligand binding, Dvl is recruited and a G-coupled protein is also recruited, which subsequently activate the phospholipase C, whose role consists in the cleavage of phosphatidylinositol-4, 5-bisphosphate (PIP2) into inositol-1, 4, 5-trisphosphate (IP3) and diacylglycerol (DAG). The IP3 diffuses in the cytoplasm to induce Ca^2+^ release by cytoplasmic organelles. Ca^2+^ increase activates the Ca^2+^ -dependent kinases.

Wnt signaling is also an ancient and evolutionarily preserved pathway. Wnt proteins are secreted glycoprotein ligands that bind Frizzled transmembrane receptors located at cell membrane level. There are more than 19 Wnt proteins and 12 Frizzled receptors. There are two types of Wnt signaling pathway, the canonical Wnt/β-catenin cascade, and the non-canonical or β-catenin-independent signaling cascade (Kusserow et al., 2005). Initially, the ligands were classified as canonical (Wnt-1, −2, −3, −8a, −8b, −10a, and −10b) or non-canonical (Wnt-4, −5a, −5b, −6, 7a, −7b, and −11), according to the kind of signal activated upon their binding to the receptors (Siar et al., 2012). Some ligands indeed are more related to the type of activation (canonical or not), while some others can trigger Wnt signaling in a β-catenin-dependent or independent manner, according to the pathophysiological context. Wnt5a, for example, was early classified as non-canonical signal, but it can both activate and repress Wnt/β-catenin signaling during embryonic development and cancer development (Sato et al., 2010; van Amerongen et al., 2012). Studies on Wnt5a highlighted two important key points: i. the two cascades are not activated together, and ii. the co-receptors involved are different, i.e., ROR1/2 for the non-canonical signaling and the low-density lipoprotein receptor-related protein family (LRP5/6) for the β-catenin-related signal (Sato et al., 2010; van Amerongen et al., 2012). Indeed, Frizzled receptors are coupled to co-receptors, such as LRP5/6, ROR2, NRH1, Ryk, and PTK7. LRP5/6 is involved in the canonical signaling, where β-catenin is sequestrated by a destruction complex made of the Axin scaffold protein associated with APC (adenomatous polyposis coli), GSK-3β (glycogen synthase kinase 3β), and CK1 (casein kinase). CK1 and GSK-3β sequentially phosphorylate β-catenin at serines 45, 33, 37 or threonine 41 (Yost et al., 1996; Amit et al., 2002). This cascade of phosphorylation triggers ubiquitylation of β-catenin by βTrCP (an E3 ligase) and its subsequent proteasomal degradation. When the ligand binds to the frizzled receptors, its coreceptors LRP5/6 recruits the Disheveled (Dvl) protein, which in turn binds to Axin and GSK-3 proteins, leading to the disassembling of the destruction complex, the release of β-catenin and its nuclear localization (Salic et al., 2000). In the nucleus, β-catenin interacts with LEF/TCF transcription factors and other transcriptional activators to trigger activation of Wnt target genes (Figure 2). The canonical Wnt signaling can be modulated at different levels: (i) Inhibitors or antagonists of the ligand/receptors, such as Dickkopf (Dkk) proteins, secreted frizzled-related proteins (sFRPs), and WNT inhibitory factor 1 (WIF1); (ii) negative feedback through phosphorylation of Axins proteins (Axin 1 and Axin 2) by GSK-3β. There are several β-catenin-independent Wnt signaling pathways all related to a specific co-receptor or other key elements. One of them is the planar cell polarity (PCP) pathway that is mainly active in epithelial and mesenchymal cells, being involved in tissue polarization. The spatio-temporal organization of the pathway is not so clear; there are at least two complexes involved in Wnt-PCP located on adjacent cells, on the distal and the proximal membrane, respectively. Core components on the distal membrane consist in Frizzled and the scaffold partners Dvl, Diego and Flamingo. The counterpart on the proximal membrane involved Van Gogh, Prickle, and Flamingo scaffolds (Vladar and Königshoff, 2020). Although the two complexes are interconnected, a simple presentation of the signal transduction after ligand binding on Frizzled receptors shows the recruitment of Dvl, Diego and Flamingo and the formation of a protein platform triggering the activity of Rho family GTPase proteins to regulate actin organization and cytoskeleton dynamics (Figure 2; Siar et al., 2012; Vladar and Königshoff, 2020). Another well-known β-catenin-independent pathway is the Wnt/Ca^2+^, which controls the levels of intracellular Ca^2+^ (Figure 2). Like the two afore mentioned cascades; Dvl is also recruited after ligand biding, but in the meantime a G-coupled protein is also recruited, which subsequently activate the phospholipase C, whose role consists in the cleavage of phosphatidylinositol-4,5-bisphosphate (PIP2) into inositol-1, 4, 5-trisphosphate (IP3) and diacylglycerol (DAG). The IP3 diffuses in the cytoplasm to induce Ca^2+^ release by cytoplasmic organelles. Ca^2+^ increase activates the Ca^2+^ -dependent kinases, such as protein kinase C (PKC), Calcium-calmodulin dependent kinase II (CamKII), and Calcium/calcineurin (CaCN). DAG also participates to the direct activation of PKC (Kusserow et al., 2005; Baksh et al., 2007; Jeong et al., 2020; Vladar and Königshoff, 2020).

### The Wnt Signaling in MSCs

The role of Wnt signaling in the control of MSC biology is well documented. Transcriptomic and proteomic approaches, such as flow cytometry, ELISA, Western immunobloting, and mass spectrometry, showed in MSCs the enrichment in both canonical and non-canonical Wnt pathway components (Kuljanin et al., 2017). Using phosphospecific antibodies, we observed that Ser33/37/Thr41-phospho β-catenin (inactive) is totally absent in MSC cell lysate, thus suggesting that the Wnt/β-catenin is fully active in MSCs (Takam Kamga et al., 2016b; Wang et al., 2019). The requirement of a functional β-catenin-independent Wnt signaling, such as Wnt/Ca^2+^, Wn/Jnk, Wnt/Ryk, Wnt/Ror2, was also described in MSCs (Qiu et al., 2011; Qu et al., 2013; Jeong et al., 2020). Overall, the activation of the pathway plays a critical role in cell fate decisions, notably for MSC proliferation, self-renewal and differentiation. In particular, Wnt signaling modulation in MSCs is widely investigated to fully exploit regenerative properties of MSCs in different research fields, such as bone, lung, and heart biology (Volleman et al., 2020). The canonical Wnt/β-catenin pathway sustains proliferation and renewal of MSCs; therefore, the use of pharmacological modulators of the pathway has brought several informations. The activation of the canonical Wnt/β-catenin pathway with lithium chloride or exogenous ligands, such as Wnt1 and Wnt3a, promotes MSC expansion by maintaining their clonogenic properties, but inhibits osteogenic, and adipogenic commitment (Liu et al., 2009, 2011; Jothimani et al., 2020). One key mechanism of the suppressive role of Wnt/β-catenin on adipogenesis is the reduced expression of adipogenic transcription factors CCAAT/enhancer binding protein alpha (C/EBPalpha) and peroxisome proliferator-activated receptor gamma (PPARgamma) (Ross et al., 2000; Yuan et al., 2016). However, the use of Wnt/β-catenin inhibitors, such as Quercitin, reduce MSC proliferation and multipotency by favoring their osteogenic commitment and inhibiting both the chondrogenic and the adipogenic differentiation (Qu et al., 2013; Narcisi et al., 2015; Jothimani et al., 2020; Volleman et al., 2020). This model failed to explain the positive contribution of canonical Wnt in bone homeostasis *in vivo* (Wagner et al., 2020). Liu et al. suggested a role for Wnt/β-catenin activation levels; in fact, they observed a promoting effect with low concentrations of Wnt3a during osteogenic differentiation, through the regulation of key transcription factors such as RUNX2 and Osterix (Osx), while higher concentrations suppressed both osteogenesis and adipogenesis (Gaur et al., 2005; Liu et al., 2009). As for quercitin-mediating promotion of osteogenesis, increased Ca^2+^ signaling was also observed upon quercitin treatment, suggesting that osteogenic switch could be modulated by the balance between canonical and non-canonical signaling. In fact, a tight crosstalk between canonical and non-canonical Wnt leads to functional antagonism during osteogenic differentiation (Baksh et al., 2007), and osteogenic suppression induced by Wnt1 and Wnt3a is correlated with reduced Ror2/JNK levels (Gaur et al., 2005; Liu et al., 2009). Therefore, these studies proposed a binary view where the activation of Wnt/β-catenin through exogenous ligands, such as Wnt3a, may suppress both osteoblastic gene expression and MSC osteogenic differentiation with decreased matrix mineralization, while the activation of the non-canonical pathway has an opposite effects (Boland et al., 2004; Jothimani et al., 2020). Moreover, the activation of canonical pathway suppresses the non-canonical pathway and vice versa. Therefore, higher concentrations of Wnt3a suppresses osteogenesis by competing with non-canonical ligands. For instance, Wnt5a stimulates osteogenesis through the Wnt/ROR2/JNK signaling by competing with Wnt3a-mediated Wnt/β-catenin. Consequently, quercitin switches the balance toward non-canonical signaling, while Wnt3a or Wnt1 switch it toward Wnt/β-catenin cascade (Baksh et al., 2007). A role for canonical and non-canonical Wnt was also observed during motility and migration processes. Some authors used lentiviral constructs to enforce the expression of β-catenin or ROR2 in MSCs. They observed that β-catenin or ROR2 upregulation induces either nuclear β-catenin accumulation or the activation of Wnt5a/JNK and Wnt5a/PKC pathways, belonging to the canonical Wnt and non-canonical Wnt5a/ROR2 pathways, respectively (Liu et al., 2009; Cai et al., 2014).

## MSC-Derived Notch and Wnt Signaling Pathways in Hematopoiesis

Hematopoiesis is the process of blood cell formation through the proliferation and differentiation of HSCs and progenitor cells into specialized cells belonging to lymphoid and myeloid lineages (Orkin and Zon, 2008). Activation of Notch and Wnt signaling pathways is essential for the maintenance of HSCs (Bigas et al., 2010). Pharmacological and loss- or gain-of-function approaches have been useful strategies to investigate the role of Notch and Wnt signaling pathways in hematopoiesis. The retroviral expression in HSC/progenitors cell-enriched populations of active forms of Notch receptors, Notch target genes or β-catenin increases the pool of cells with repopulating capacities, such as Lin- cord blood cells, CD34^+^ CD38- and mouse KLS (c-Kit^+^ Sca1^+^ Lin-) cells (Varnum-Finney et al., 2000; Kunisato et al., 2003; Reya et al., 2003; Vercauteren and Sutherland, 2004). Accordingly, the addition of exogenous ligands of the two pathways, such as Jagged-1 or DLL-1 (Notch signaling), and Wnt3a (canonical Wnt signaling), to cultures of purified primitive human blood progenitors induces self-renewal, survival and expansion of stem cells provided with pluripotent repopulating capacity in mouse models (Karanu et al., 2000; Willert et al., 2003; Delaney, 2005). Our and other groups have thoroughly described the expression of Notch and Wnt signaling in MSCs (Kamdje et al., 2011; Kamdje et al., 2012; Takam Kamga et al., 2016b), but other MSC-derived stromal components, including osteoblasts, and endothelial cells, can be the source of paracrine Wnt and Notch signaling in the BM (Nemeth et al., 2009; Wang et al., 2016). Moreover, MSCs can reconstitute the complete human BMME in irradiated mice (Muguruma et al., 2006) and therefore improve HSC engraftment following transplantation (Zhao et al., 2019). MSCs, expressing Notch and Wnt components, represent a major source of exogenous Notch or Wnt ligands that are involved in HSC fate. Using both co-culture and repopulation assay in SCID mice, Kadekar et al. observed that MSCs supported HSC expansion by preventing the apoptosis of primitive HSCs through a higher expression of β-catenin, DLL-1, Jagged1, Hes1, Notch1, and cleaved Notch1 (NICD1) (Kadekar et al., 2015). Similarly, several works have clearly showed the enhanced expression of Notch and Wnt signaling in both co-cultured MSCs and hematopoietic progenitors leading to proliferation and maintenance of HSCs on MSC feeder layer (Kim et al., 2009, 2015a, 2018; Kikuchi et al., 2011). Interestingly, increased levels of Notch components in MSCs resulted from the activation of β-catenin pathways. Growing evidence supports a model where HSC-MSC co-culture leads to higher level of β-catenin in MSCs, whose gene transactivation may lead to Jagged1 expression, which in turn acts as paracrine ligand to trigger activation of Notch signaling in HSCs. Wnt/β-catenin signals in MSCs enhance HSC self-renewal by inducing the crosstalk of Wnt-Notch signals in the HSC niche (Kim et al., 2009; Oh, 2010; Kadekar et al., 2015). Therefore, the canonical Wnt signaling is significantly required by stromal cells (Jeong et al., 2020). Excess of canonical Wnt signaling in HSCs impairs the function of HSCs and their multilineage progenitors (Scheller et al., 2006); as previously mentioned, this could be explained by the competition between canonical and non-canonical Wnt cascades. Higher levels of canonical signaling suppress the non-canonical one. Activation of the non-canonical Wnt, with Wnt5a and the co-receptor Ryk, leads to HSC quiescence, whereas Wnt3a, the canonical ligand, supports HSC proliferation (Liu et al., 2011; Jeong et al., 2020). The involvement of the non-canonical cascade may explain why Notch and Wnt pathways are also involved in mediating adhesion and migration of HSCs. The aforementioned work by Kadekar et al. showed enhanced levels of Wnt/Notch components as well as migration and adhesive properties in HSCs cultured on MSCs (Kadekar et al., 2015). The crosstalk of Notch or Wnt pathways with stromal cell-derived factor-1 (SDF-1)/CXCR4 axis is well described and may be responsible for their influence on HSCs migration and adhesion (Tamura et al., 2011; Kadekar et al., 2015). Duryagina et al. (2013) observed that Jagged1 expression by MSCs induces the release of SDF-1, thus supporting proliferation, migration, and adhesion of CD34^+^ progenitors, resulting in the increase of cobblestone area-forming cells and long-term culture-initiating cells (LTC-ICs). Notch and Wnt signaling are involved not only in the maintenance of HSCs, but also in T-cell differentiation. Delaney et al. observed that the treatment of CD34^+^ CD38^–^ cord blood progenitors with low density of DLL1 enhanced generation of NOD/SCID repopulating cells, while high density of DLL1 induced a switch toward lymphoid rather than myeloid lineage (Delaney, 2005). However, higher levels of Notch pathway preferentially support T cell differentiation by stimulating the common lymphoid progenitor toward T-cell rather than B-cell lineage. Precursor cells engineered to express NICD1 and engrafted in immunodeficient mice give rise to T-cell populations only. Conversely, silencing Notch activity leads to the onset of B-cell progeny (Wilson et al., 2001). Similarly, MSCs may support T-cell differentiation of co-cultured precursor cells when forced to express Notch receptors (Notch1 and Notch2) and ligands (Jagged1 and DLL1) (Felli et al., 1999; Aster, 2005; Vacca et al., 2006). During this process, the type of the ligands expressed by stromal cell is crucial. Some MSC cell lines, such as OP9, expressing different Notch ligands, showed that MSC-derived DLL4 supports both αβ- and γδ-lineage differentiation, while MSC-derived Jagged1 supports TCR-αβ, but not TCR-γδ development and MSC-derived Jagged2 mainly supports γδ T cell differentiation at the expense of αβ T cells (Van de Walle et al., 2013). Assays with OP9 cell line were also useful to understand the contribution of stromal cell-derived Wnt signaling to T-cell development. Famili et al. (2015) engineered OP9 cells to conditionally express either Wnt3a or Wnt5a. They observed that low density of the canonical Wnt ligands accelerates T-cell proliferation and maturation, while higher levels of the signal blocks T-cell development and favors alternative lineages. In parallel, *in vitro* experiments showed no effect of the non-canonical Wnt ligand (Wnt5a). During the T-cell switch, thymic stromal cell-derived Wnt signaling influence T-cell expansion and maturation by controlling the activation of transcription factors of the T-cell factor/lymphoid enhancing factor (Tcf/Lef) family (Schilham et al., 1998; Staal et al., 2001; van Loosdregt et al., 2013). This is associated with defective final differentiation and reduced thymocyte number in mice, either expressing the inhibitor of β-catenin and Tcf (ICAT) or resulting deficient for canonical Wnt ligand, such as Wnt1 (Mulroy et al., 2002; Pongracz et al., 2006). Famili et al. (2015) observed that in the co-co-culture setting with OP9 cell line or in mouse models, low levels of β-catenin signaling supports T-cell development, whereas higher activity of canonical and non-canonical Wnt preferentially favors myeloid and B-cell developments. Notably, the regulation of hematopoiesis by canonical Wnt requires the physical contact between MSCs and hematopoetic cells (Ichii et al., 2012; Famili et al., 2015). MSCs and stromal cell mediated Wnt signaling is therefore required at all steps of the hematopoiesis, being a decisional factor for lymphoid and myeloid switch. Concerning myeloid lineage, the role of Notch and Wnt pathways is not well-defined compare to the lymphoid counterpart. For example, myelopoiesis has been associated with low levels of Notch signaling (de Pooter et al., 2006; De Obaldia et al., 2013). However, this view may underestimate the complexity of Notch contribution to myeloid lineage development. Notch involvement in myeloid differentiation is certainly lower, as compared to lymphopoiesis (De Obaldia et al., 2013); nevertheless, the fine tuning of Notch levels is fundamental for myeloid cell development. The role of Notch could be phase-dependent during myeloid cell generation (Fehon et al., 1991). For instance, constitutive Notch activation in 32 myeloid progenitor cells led to self-renewal of myeloid precursors and inhibition of granulocytic differentiation (Milner et al., 1996). The same results were also achieved in HL-60 cell line, which failed to undergo ATRA-mediated differentiation when genetically enforced to express NICD1 (Carlesso et al., 1999). Conversely, Jagged1 may inhibit proliferation of macrophage progenitors (Masuya et al., 2002; Kim et al., 2009; Kadekar et al., 2015) and Notch pathway seems to be involved in the differentiation of mature myeloid cells (Fehon et al., 1991). The complexity of Notch contribution to myeloid lineage could arise from the level of the pathway activation. Using *ex vivo* systems for the expansion of cord blood CD34 + CD38- HSC progenitors, DLL-1 at lower density was capable of enhancing the generation of CD34^+^ cells as well as CD14^+^ and CD7^+^ cells, consistently with early myeloid and T-cell differentiation, respectively. However, culture with higher amounts of DLL-1 induced apoptosis of CD34^+^ precursors, thus resulting in decreased cell numbers, without any effects on the generation of CD7^+^ cells (Delaney, 2005). A minimal activity of Notch could be necessary for the maintenance of myeloid progenitors, while higher activation could induce cell differentiation. Again, the source of paracrine ligands that trigger Notch activation in myeloid progenitors might be stromal cells. Indeed, primitive (CD34^+^ CD38^–^ Lin^–^), and intermediate (CD34^+^ CD38^+^ Lin^–^) HSCs cultured on MSCs expressing Jagged1 or DLL-1 showed enhanced self-renewal properties associated with increased expression and activation of Notch1. This suggests that in the BM niches MSCs provide exogenous Notch ligands necessary for the maintenance of myeloid progenitor pool and Jagged1 expression is the consequence of Wnt/β-catenin activation, thus suggesting a role for Wnt-Notch cross-talk in myelopoiesis (Fernández-Sánchez et al., 2011). In parallel, thanks to *in vitro* colony-replating assays, Nteliopoulos et al. observed that canonical and non-canonical Wnt-3 can stimulate proliferation of myeloid progenitors and impair IL-3-induced differentiation into myeloid populations (Nteliopoulos et al., 2009). As MSCs are a source of Wnt ligands, we can hypothesize that stromal cells may support the self-renewal of myeloid progenitors through the release of Wnt ligands (Toni et al., 2006). However, there are a few studies addressing the role of MSC-derived Wnt signaling in myeloid counterpart. Most data arise from studies on myeloid malignancies and will be discussed in the next section.

## MSC-Derived Notch And Wnt Signaling in Leukemia

### Notch in Leukemia

Several studies have addressed the role of Notch in leukemic diseases (Table 1). Early association between Notch and hematopoietic malignancies was shown in T-ALL, where more than 50% of patients have activating mutations of Notch signaling, thus representing the first gene aberration in T-ALL (Weng et al., 2004). Notch mutations in T-ALL mainly target the HD or the PEST domains. By sequencing the heterodimerization domain of NOTCH1 in mouse models of T-ALL, O’Neil (2006) found that more than 74% of the tumors harbored activating mutations in Notch1. Mutations in HD domain induce a constitutive, ligand-free activity of the receptors. The second hotspot of mutations is the PEST domain targeting NICD to ubiquitination-mediated proteolysis. The mutation in the PEST domain determines the lack of degradation of the active form of the receptors, thus leading to a constitutive activity of the pathway (Weng et al., 2004). In nude mouse models of T-ALL, tumor establishment correlated with Notch1 mutation (Lin, 2006). The importance of Notch activation for T-ALL cell survival has raised the use of gamma-secretase inhibitors (GSIs). T-ALL cells are highly sensitive to different GSIs (Grosveld, 2009; Real and Ferrando, 2009; Baratta, 2019) as well as to other Notch inhibitors, such as Notch transcription factor inhibitors (Moellering et al., 2009) and Notch blocking antibodies (Wu et al., 2010). Besides Notch1, higher levels of Notch3 were found in T-ALL cells, and its genetic inhibition through siRNA led to growth inhibition and apoptosis (Masiero et al., 2011). Constitutive activation of Notch is also a hallmark of B-cell CLL. Notch activating mutations occur essentially in the PEST domain of Notch receptors and are associated with a shorter overall survival (Willander et al., 2013). Rosati et al. (2013) found high expression of Notch1, Notch2, Jagged1, and Jagged2 in CLL correlated with higher activation of the pathway. This activation is further increased in CLL cells that are resistant to spontaneous apoptosis in *ex vivo* culture. Accordingly, our group demonstrated that Notch inhibition, through GSIs or blocking antibodies, induces CLL apoptosis, and sensitizes leukemia cells to treatment with chemotherapeutic agents (Kamdje et al., 2012). Except in T-ALL, Notch mutations are very rare in other leukemia types, where its role is either well defined or quite controversial (Liu et al., 2013). In B-cell acute lymphoblastic leukemia (B-ALL), Notch1 mutation was not observed, but a tumor suppressor role of the pathway was suggested (Morimura et al., 2000; Zweidler-McKay et al., 2005). Notch seems to be epigenetically silenced in B-ALL, since Notch3, Jagged1, Hes2, Hes4, and Hes5 are frequently hypermethylated in leukemia B-cell lines and primary B-ALL cells. Restoration of Hes5 expression by lentiviral transduction resulted in growth arrest and apoptosis in Hes5-negative B-ALL cells (Kuang et al., 2013). Activation of the pathway induces growth arrest and apoptosis in B-ALL cells (Morimura et al., 2000; Zweidler-McKay et al., 2005; Kuang et al., 2013). Putting in the context of anti-leukemic treatment, epigenetic analysis of blast cells collected from B-ALL patients along the course of the disease revealed that the methylation pattern of Notch receptors’ genes changes according to the disease step. It was observed that Notch genes receptors are highly methylated at diagnosis, less methylated upon drug treatment and became hypermethylated in relapsed patients (Takam Kamga et al., 2019a). These observations suggested that the methylation status of Notch genes might be relevant for drug response. This is strengthened by the results obtained in non-leukemic systems where evidence of epigenetic modulation of Notch genes in cancer cells treated with chemotherapeutic agents like 5-fluorouracil and cisplatin was demonstrated (Maeda et al., 2014). Collectively these data support further research to unravel the role of epigenetic silencing of Notch in leukemia disease. Studies in solid cancers have also reported that Notch genes are the targets of several miRNA (or vice-versa) involved in drug resistance including miR-1, miR-200, miR-34 etc. (Ji et al., 2009; Li et al., 2009). Consistently recent studies have provided the evidence that the BM-microenvironment transfer miRNA in leukemia cells, supporting cell survival (Liu et al., 2015; Ganesan et al., 2019).

**TABLE 1:** Roles of Notch and Wnt signaling pathways in leukemia.

		Leukemia cell-derived Notch/Wnt signaling	MSC-derived Notch/Wnt signaling
AML	Biomarkers	– Higher expression and activation of Notch signaling components is associated to poorer prognosis in AML (Xu et al., 2011; Sliwa et al., 2014; Takam Kamga et al., 2019a). – High activation of Wnt/β-catenin is associated to shorter survival (Khan and Bendall, 2006; Griffiths et al., 2010).	– Overexpression of Notch1 and Jagged1 in AML-MSCs (Takam Kamga et al., 2016a). – Overexpression of Wnt molecules in AML-MSCs (Takam Kamga et al., 2016b).
	Oncogene	– Notch/Jagged1 expression and activation in acute promyelocytic leukemia (APL) supports leukemia cell growth (Grieselhuber et al., 2013). – Activation of Wnt/β-catenin/TCF/LEF pathway supports growth of leukemia cells (Khan and Bendall, 2006). – Epigenetic modification of Wnt inhibitors in AML (Griffiths et al., 2010).	– Notch signaling is required for β-catenin-mediated oncogenesis in mouse models of AML (Kode et al., 2014). – MSC-derived Notch signaling supports growth and survival of leukemic cells (Takam Kamga et al., 2016b). – MSC-derived Notch signaling supports growth and survival of leukemic cells (Takam Kamga et al., 2016a).
	Tumor suppressor	– Enforced expression of Notch receptors in AML inhibits leukemia cell growth and survival (Kannan et al., 2013; Lobry et al., 2013)	
	Mediator of drug resistance		– MSC-derived Notch signaling reduces apoptosis in AML treated with chemotherapeutic agents (Takam Kamga et al., 2016a). – Stromal cell-derived Wnt signaling reduces apoptosis in AML treated with chemotherapeutic agents (Takam Kamga et al., 2016b)
B-ALL	Biomarkers	– Higher expression and activation of Notch signaling is observed in refractory patients (Kamdje et al., 2011; Takam Kamga et al., 2019b). – Wnt ligands and receptors are overexpressed in B-ALL cells (Khan et al., 2007). – Overexpression of LEF1 predicts poor outcomes (Kühnl et al., 2011)	
	Oncogene	– Epigenetic inactivation of Notch in B-ALL (Kuang et al., 2013). – Stimulation of Wnt/β-catenin signaling supports growth and survival of B-ALL cells (Khan et al., 2007).	– MSC-derived Notch signaling supports growth and survival of leukemic cells (Kamdje et al., 2011). – MSC-derived Wnt signaling supports growth and survival of leukemic cells (Yang et al., 2013).
	Tumor suppressor	– Activation of Notch signaling induce cell cycle arrest and apoptosis (Morimura et al., 2000; Zweidler-McKay et al., 2005; Kuang et al., 2013).	
	Mediator of drug resistance	– Notch inhibitors sensitize B-ALL cells to chemotherapy (Takam Kamga et al., 2019b). – Wnt inhibition sensitizes B-ALL to chemotherapy (Fu et al., 2019).	– MSC-derived Notch signaling reduces apoptosis in B-ALL treated with chemotherapeutic agents (Kamdje et al., 2011). – MSC-derived Wnt signaling reduces apoptosis in B-ALL treated with chemotherapeutic agents (Yang et al., 2013).
CLL	Biomarkers	– Notch activating mutation are observed in CLL patients (Willander et al., 2013). – Notch1 mutation is found in intermediate-risk patients, predicting poorer survival (Willander et al., 2013). – Higher expression and activation of Notch signaling is observed in refractory patients (Rosati et al., 2013). – Wnt5 is enriched in CLL patients (Janovska et al., 2016). – Low WNT3 expression is a signature of patient with short therapy-free survival (Janovská and Bryja, 2017).	
	Oncogene	– Activation of Notch signaling supports growth and survival of CLL cells (Kamdje et al., 2012; Rosati et al., 2013). – Lef1 is a prosurvival factor s (Willander et al., 2013). – Wnt/PCP controls migration of CLL cells (Janovska et al., 2016).	– MSC-derived Notch signaling supports growth and survival of leukemic cells (Kamdje et al., 2012). – MSC-induced accumulation of β-catenin in CLL cell supports growth and survival of leukemia cells (Mangolini et al., 2018).
	Tumor suppressor		
	Mediator of drug resistance	– Notch inhibitors sensitize CLL cells to chemotherapy (El-Gamal et al., 2014).	– MSC-derived Notch signaling reduces apoptosis in CLL cells treated with chemotherapeutic agents (Kamdje et al., 2012; Mangolini et al., 2018). – MSC-induced accumulation of β-catenin in CLL cells, supports drug resistance of leukemia cells (Mangolini et al., 2018).
CML	Biomarkers		
	Oncogene	– β-catenin is a target of BCR-ABL (Zhao et al., 2007; Tomasello et al., 2020) – Wnt1 signaling supports growth and survival of CML cells (Majeti et al., 2009).	
	Tumor suppressor	– Notch1 suppresses growth and survival of K562 cell line (Yin et al., 2009).	
	Mediator of drug resistance	– Inhibition of Wnt/β-catenin sensitizes cells to TKI (Zhang et al., 2013).	– MSC-derived Notch signaling reduced apoptosis in CML treated with chemotherapeutic agent. – MSC-derived Wnt signaling reduced apoptosis in CML cells treated with TKI (Han et al., 2013; Zhang et al., 2013).
T-ALL	Biomarkers		
	Oncogene	– Notch1 is mutated in more than 50% of patients (Weng et al., 2004). – Notch signaling drives oncogenesis and supports growth and survival of T-ALL cells (Weng et al., 2004; O’Neil, 2006). – Notch 3 supports survival of T-ALL cells (Masiero et al., 2011).	
	Tumor suppressor		
	Mediator of drug resistance	– Notch inhibition sensitizes cells to drug treatment (Grosveld, 2009; Real and Ferrando, 2009).	– MSC-derived Notch/Jagged1 signaling reduces apoptosis in Jurkat cell line treated with chemotherapeutic agents (Yuan et al., 2013). – MSC-derived Wnt signaling reduces apoptosis in ALL cell treated with chemotherapeutic agents (Yang et al., 2013).

Our group has recently shown that human BM MSCs, through Notch activation, protect B-ALL cells from apoptosis induced by chemotherapeutic agents; in fact, Notch signaling inhibition abrogates the protective role of human BM MSCs toward B-ALL cells (Kamdje et al., 2011), thus highlighting the contribution of the BMME in Notch signaling. In myeloid malignancies, the role of Notch is still matter of investigation. In chronic myeloid leukemia (CML), Notch emerges as tumor suppressor gene rather than oncogene, although still poorly investigated. Yin et al. (2009) observed that overexpression of Notch1 active form in the CML cell line K562 significantly inhibits cell proliferation, while knocking-down the pathway through the expression of a dominant negative of RBP-jk promotes colony-forming activity. In acute myeloid leukemia (AML), the role of Notch remains controversial: Kannan et al. (2013) described Notch expression and activation in *ex vivo* AML cell samples and AML cell lines, but weak activation of the pathway, as demonstrated by the low expression level of the Notch target genes. Similarly, Lobry et al. (2013) described epigenetic silencing of Notch target genes in AML; consistently, they demonstrated that the reactivation of Notch signaling induced apoptosis and differentiation of leukemia blast cells into mature cells. These results are consistent with the anti-leukemic role of demethylating/hypomethylating agents azacytidine or decitabine in AML (DiNardo et al., 2018; Leung et al., 2019). However, our and other groups found that Notch activation is not homogenous within AML samples and cell lines (Tohda and Nara, 2001; Sliwa et al., 2014; Czemerska et al., 2015). In the study by Tohda and Nara (2001) 6 cell lines out of 8 and 40% of AML fresh samples showed active forms of Notch1 receptors. Some observations suggest that Notch expression and activation levels in AML could be correlated with the molecular background of each samples or the FAB subgroup (Tohda and Nara, 2001; Salat et al., 2008; Grieselhuber et al., 2013; Sliwa et al., 2014; Czemerska et al., 2015; Takam Kamga et al., 2019a). For example, ETO in association with RBP-jk inhibits the expression of Notch target genes, while the leukemogenic fusion protein AML1/ETO is devoid of this repressive activity (Salat et al., 2008). Grieselhuber et al. (2013) identified Notch expression and activation in acute promyelocytic leukemia presenting the PML-RARα rearrangement. However, Notch pathway activation has been observed mostly in more immature AML subtypes and was associated with bad prognosis, as patients with hyper-expression of Notch1 displayed poorer overall survival (Xu et al., 2011; Sliwa et al., 2014; Takam Kamga et al., 2019a). Notably, in a recent study we found that less mature AML subtypes (M0-M1) expressed high levels of all the four receptors (Notch1–4) and some ligands (Jagged2, DLL-3), whereas adverse cytogenetic risk groups overexpressed Notch3, Notch4, and Jagged2 as compared to good cytogenetic risk patients. Accordingly, univariate and multivariate analysis confirmed a longer overall survival for patients presenting low expression of Notch4, Jagged2, and DLL3 on leukemia cells at diagnosis (Takam Kamga et al., 2019a).

### Wnt Signaling in Leukemia

Wnt pathway deregulation is a common feature of leukemia. In lymphoid malignancies, such as ALL, CLL non-canonical and canonical Wnt pathway-related genes and proteins are over-expressed in lymphoid tumor cells, thus resulting prone to apoptosis upon interference with the pathway including β-catenin inhibition (Rosenwald et al., 2001; Lu et al., 2004; Janovská and Bryja, 2017). Consistently, over-expression of LEF-1 mRNA is a hallmark in ALL and CLL patients with poor prognostic. The constitutive activation of the pathway deregulation can result from gene mutation (Tomasello et al., 2020), but also from epigenetic modifications. In CLL for example, Next generation sequencing of samples from patients confirmed that 40% of patients harbors somatic mutations in Wnt pathway components (*WNT1*, *WNT10A*, *DKK2*, *RSPO4, FZD5, RYK*) (Wang et al., 2014). Studies have indicated a crosstalk between molecular aberrations and epigenetic activation of the pathway, acting in a concerted manner to interfere with Wnt inhibitors while promoting Wnt agonists or activators. Consistently the promoter of genes coding for Wnt pathway inhibitors including *WIF1, DKK3, APC, SFRP1, SFRP2, SFRP4*, and *SFRP5* are frequently hypermethylated and consequently downregulated in samples from CLL and ALL (Roman-Gomez et al., 2004; Martin et al., 2008; Rahmatpanah et al., 2009). It is worthy to mention that the tumor suppressor gene *APC* could also be the target of epigenetic modification. In T-ALL, the promoter of APC is methylated in about 50% of cases and correlates with β-catenin over-expression (Matsushita et al., 2006). In B-ALL cell lines and primary B-ALL cells, the Wnt pathway is activated by over-expression of Wnt proteins and receptors (Wnt-2b, Wnt-5a; Wnt-10b, Wnt-16b; FZD7; FZD8) and their stimulation with Wnt-3a increases the survival and proliferation of these cells (Khan et al., 2007). Similarly to what is observed in CLL, the hyperactivation of the pathway is due at least in part to the hypermethylation of the Wnt inhibitors (Kong et al., 2018). Concerning myeloid malignancies, Zhao et al. found that β-catenin deletion causes a reduction in the ability of mice to develop BCR-ABL-induced CML (Zhao et al., 2007). Indeed, stabilization and nuclear localization of β-catenin is a direct consequence of the BCR-ABL (Tomasello et al., 2020). As a consequence, the treatment of CML stem/progenitor cells with β-catenin inhibitor ICG001 reduces cell survival and proliferation by sensitizing cells to tyrosine kinase inhibitors (TKI). Interestingly, the addition of purified Wnt1 activates β-catenin and protects CML cells from TKI treatment, thus confirming the important role of Wnt pathway in maintaining CML stem cells (Zhang et al., 2013). In AML, our and other groups have observed an enrichment in Wnt components in AML primary cells compared to normal hematopoietic progenitors, although the expression of the Wnt components was not homogenous across samples (Majeti et al., 2009). Interestingly, β-catenin was enriched in high-risk patients; subsequently, we observed that patients presenting higher activation of the pathway had shorter progression free survival (Takam Kamga et al., 2020). The pivotal role for Wnt pathway in AML pathogenesis is also supported by studies in which cells transfected with AML-associated translocation products (PLZF-RARA and AML1-ETO) display activation of pakoglobin, a homolog of β-catenin. This induction is followed by the transactivation of TCF/LEF transcription factors and the increase in the proliferation and survival of murine hematopoietic progenitor cells (Khan and Bendall, 2006; Griffiths et al., 2010). In fact, the constitutive activation of Wnt signaling in AML may not result from β-catenin mutation, but from Flt3 hyperexpression leading to Akt-mediated phosphorylation and GSK-3β inactivation, with β-catenin stabilization (Brandts et al., 2005; Román-Gómez et al., 2007; Valencia et al., 2009). In accordance with the pro-oncogenic role of Wnt in AML, β-catenin down-regulation in AML cell lines and *ex vivo* cells through shRNA or pharmacological inhibitors, such as quercitin IWP-2, Niclosamide and PNU-74654, decreases their proliferation rate *in vitro* and homing as well as their engraftment after xenotransplantation (Toni et al., 2006; Gandillet et al., 2011; Takam Kamga et al., 2020). Interestingly, the Wnt inhibitors quercetin induced pronounced apoptosis in AML, *in vivo* and *in vitro* in part by its demethylating activity (Maso et al., 2014; Alvarez et al., 2018). In fact, in AML, the use of demethylating agents such as Decitabine decreased methylation status of Wnt antagonist including SFRP1, HDPR1, and DKK3, providing evidence that activation of the pathway resulted from an epigenic silencing (Li et al., 2014). Similarly to CLL, in AML the promoter of genes coding for Wnt antagonists (sFRP1, sFRP2, sFRP4, sFRP5, DKK1, and DKK3 etc.) are frequently methylated predicting poor outcome in patients (Jost et al., 2008; Valencia et al., 2009).

### The Role of MSCs in Leukemia

As previously discussed, several studies have reported a supportive and protumorigenic role for MSCs toward different leukemia subtypes, including AML, B-ALL, CLL, CML, and T-ALL (Lee et al., 2019). A comparison of MSCs isolated from myeloid and lymphoid leukemia environment compared to MSCs isolated from healthy donors revealed that stromal cells are the sites of deep molecular changes involving modulation of the expression and/or secretion of cytokines, chemokines, adhesion molecules, and extracellular matrix molecules such as SDF-1/CXCR4, CD44. These modifications are thought to improve MSCs-mediated survival and growth of leukemic cells and mainly leukemia stem/progenitors cells (Ge et al., 2011; Yu et al., 2019; Azadniv et al., 2020). MSCs have the double ability to keep leukemic stem cells in a quiescent state while promoting proliferation and growth of leukemia cells. Coculture experiments showed that MSCs supports the culture of primary leukemia cells and promote the long term survival of leukemia stem cells (Ito et al., 2015). Evidence from studies support a bidirectional crosstalk between MSCs and leukemia stem/progenitor cells. In the study of the Yu et al. (2019), they observed that MSCs co-cultured with B-ALL leukemia stem cells showed downregulation of lumican increased expression of CD44 and diverse chemokine including IL-3, IL-7, IL-10, and G-CSF. These educated MSCs were more potent to protect leukemic cells against VP-16. Similarly, in AML, CXCR4, CD44, integrins like VCAM1 or VLA-4 are activated upon the contact between AML cells and MSCs to promote resistance of leukemia cells. A treatment of AML cells with the specific CXCR4-SDF inhibitor, AMD3100 or antibodies against CD44, VCAM1, significantly sensitizes AML stem cells to treatment with chemotherapeutics, thus abrogating MSCs mediated chemoresistance and persistence of the minimal residual disease (Matsunaga et al., 2003; Tabe et al., 2007; Nervi et al., 2009; Jacamo et al., 2014). This mechanism can be translated in other leukemia as demonstrated by several studies (Konopleva et al., 2009).

### Putting Together the Contribution of MSC-Derived Notch and Wnt Signaling Pathways in Leukemia

**FIGURE 3:**
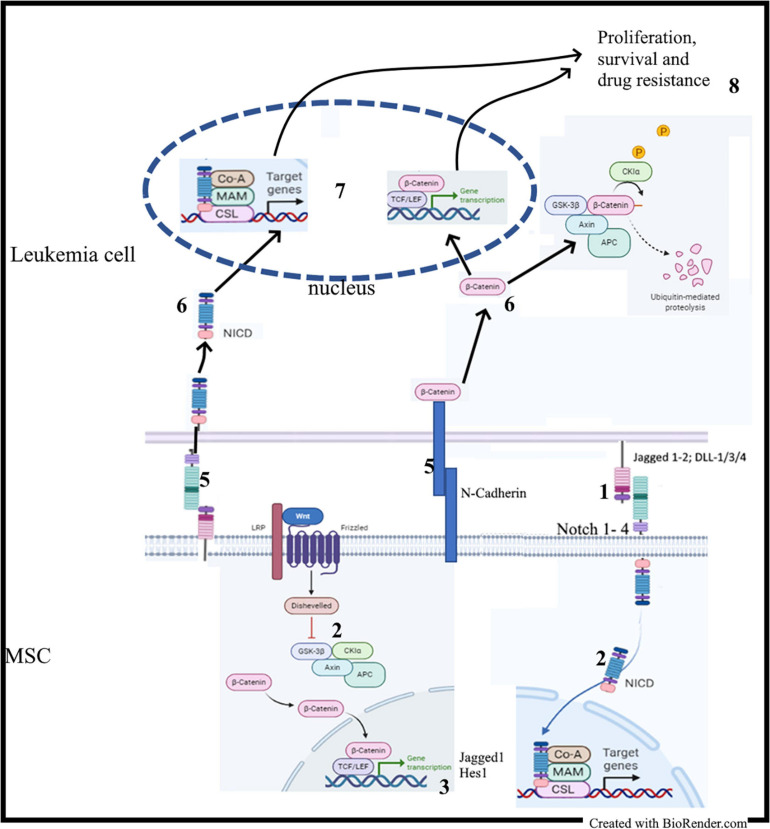
MSC-derived Notch and Wnt signaling in leukemia: (1) Contact between MSC and leukemia cells; (2) Activation of the Notch and Wnt signaling cascades in MSC; (2) Synthesis of Notch and Wnt target genes including Jagged1; (4) Upregulation of Notch1 and Jagged1 expression; (5) Activation of adhesion molecules and Notch signaling; (6) Release of NICD and stabilization of β-catenin; (7) Transactivation of Notch and Wnt target genes.

Stromal BMME promotes the survival of leukemia cells through the activation of many pathways, including Notch and Wnt signaling (Vianello et al., 2010; Kamdje et al., 2011, 2012; Tabe and Konopleva, 2015; Cai et al., 2016; Takam Kamga et al., 2016a). On the other hand, Notch and Wnt signaling are the targets of persistent modifications occurring often in parallel in the BM niche during leukemogenesis (Kode et al., 2014; Kim et al., 2015b). Therefore, analyzing MSCs isolated from leukemia samples can provide an overview of these persistent modifications involving both pathways, which eventually can be considered as a unique microenvironmental communication system, the so called Wntch pathway (Sengupta et al., 2007; Hayward et al., 2008; Takam Kamga et al., 2016a; Azadniv et al., 2020). Studies revealed that, increasing activity of Notch signaling results from an aberrant β-catenin signaling in the same stromal compartment and vice versa (Kode et al., 2014). In normal hematopoiesis, stromal β-catenin signaling induces expression of Jagged1; consequently, stromal Jagged1, and Wnt ligands induce in HSCs Notch and Wnt signaling, respectively, and support their self-renewal in a cell-to-cell contact-dependent manner (Ichii et al., 2012; Kadekar et al., 2015). The same phenomenon occurs in leukemia cells and stem cells, where studies reported higher levels of stromal Notch parallel with higher activation of the Wnt signaling (Figure 3) (Yang et al., 2013; Takam Kamga et al., 2016a, b). Therefore, Notch signaling is required for leukemic role of the canonical Wnt (Kode et al., 2014). The functional outcome of this Wnt/Notch crosstalk between MSCs and B-ALL or AML cells is the induction of leukemia cell proliferation, survival and chemoresistance. Consequently, Wnt and/or Notch inhibition through pharmacological modulators, including small molecules inhibitors (PNU-74654, Niclosamide, GSIs) and Notch blocking antibodies, may sensitize leukemia cells to drug treatment, thus abrogating the protective role of MSC monolayer (Kamdje et al., 2011; Takam Kamga et al., 2016a, 2020; Fu et al., 2019). This antileukemic role requires the production of reactive oxygen species (ROS) and the modulation of prosurvival proteins, such as mTor, NF-κB, STAT-3, and Erk (Kamdje et al., 2011; Takam Kamga et al., 2016a, b). This role observed in *ex vivo* co-culture systems was validated in mouse models of AML and B-ALL (Toni et al., 2006; Yang et al., 2013; Takam Kamga et al., 2019a, b).

The Notch-dependent role of Wnt/β-catenin was also described in CLL; in this disease, the non-canonical Wnt/PCP/ROR1 is the main activated Wnt signaling and is involved in migration of leukemic cells (Janovska et al., 2016). Constitutive activation of β-catenin is low, but this does not exclude its involvement in the pathogenesis of CLL (Lu et al., 2004; El-Gamal et al., 2014; Mangolini et al., 2018). In fact, CLL cells constitutively express Notch receptors and ligands, whereas MSCs from CLL patients show upregulated Notch receptors and ligands (Kamdje et al., 2012). Culture of primary CLL cells on primary MSCs or EL08-1D2 stromal cell line leads to Notch 2 activation in MSCs, which in turn induces activation of Wnt/β-catenin in co-cultured CLL cells. On the other hand, conditional deletion of Notch2 in MSCs prevents β-catenin accumulation in CLL cells (Kamdje et al., 2012; Mangolini et al., 2018). Again, the use of Notch inhibitors (GSIs or Notch blocking antibodies) chemosensitizes CLL cells cultured on MSCs monolayer (Kamdje et al., 2012). N-cadherin, a crucial molecule regulating migration and homing of normal hematopoietic cells, is required for the stabilization of β-catenin in co-cultured CLL cells as well as CML cells (Kamdje et al., 2012; Han et al., 2013; Zhang et al., 2013; Mangolini et al., 2018). Consequently, it represents a central mechanism involved in the crosstalk between β-catenin and adhesion molecules to mediate chemoresistance (Toni et al., 2006; Zhang et al., 2013).

In T-ALL, the role of Notch as tumor-driven mechanism has been thoroughly studied, but the influence of stroma-derived Notch signaling is necessary for leukemia cell survival (Ntziachristos et al., 2014) as well as for chemoresistance toward dexamethasone and asparaginase (Iwamoto et al., 2007; Yuan et al., 2013; Cai et al., 2016). Contact with MSCs enhances Notch1, Jagged1, and CD28 expression on T-ALL cells (Yuan et al., 2013) and promotes leukemia cell homing into BM niche in xenotransplantation models; on the other hand, IL-6, SCF, HIF-1α, VEGFα, and Notch ligand Jagged1 is overexpressed in stromal cells (Wang et al., 2016). This aberrant stromal Notch activation negatively regulates CXLC12 in stromal cells, thus hampering their supportive functions toward HSCs and promoting preferentially T-ALL cell development. By contrast, Notch blockade reverts leukemia-associated abnormal blood lineage distribution, thrombocytopenia, and osteoblast functions (Wang et al., 2016). In co-culture, Jagged1 expression on MSCs induces drug resistance in the T-ALL cell line Jurkat, which is prevented by anti-Jagged1 neutralizing antibodies (Yuan et al., 2013). Similarly, the specific β-catenin inhibitor XAV939 may suppress T-ALL cell resistance to cytarabine, thus suggesting that Wnt/Notch cross-talk can be involved in T-ALL and deserves additional investigation (Yang et al., 2013). Overall, the use of Notch or Wnt inhibitors in coculture experiments, impeded increased activity of Notch and Wnt signaling both in leukemia and stromal cells, thus suppress enhancing leukemia survival and drug resistance. The challenge consists in the availability of efficient and safe Notch and Wnt inhibitors.

### Pharmacological Strategies to Interfere With Wnt/Notch Signaling in Cancer

Given the importance of Wnt and Notch pathways in cancer development and chemoresistance, numerous pharmacological inhibitors have been developed both as research tools but also as future anticancer drugs (Rizzo et al., 2008; Ntziachristos et al., 2014; Takebe et al., 2015). Inhibitors are designed to target specific steps of the signaling cascade such as ligand-receptors interaction, receptors processing, cytoplasmic effectors, and the formation of transcription complexes (Table 2). In the Wnt cascade, inhibitors of ligand-rectors interaction have been developed with regards to natural antagonists of the pathways. Notably, recombinant DKK (DKK-1-4) and SFRP (SFRP1-4) proteins have been developed and used in preclinical experiments to inhibits Wnt signaling in AML, multiple myeloma, and other hematological malignancies (Toni et al., 2006; Chim et al., 2007). Recombinant antibodies directed against Frizzled have also been successfully developed (Pavlovic et al., 2018). Quercetin (a polyphenol) and Niclosamide (an anthelminthic) are both capable to kill leukemia cells and stem cells at least in part by interfering with LRP5/6 (Lu et al., 2011; Maso et al., 2014; Alvarez et al., 2018; Takam Kamga et al., 2020). The post-tranlational addition of porcupine on Wnt ligand is required for the secretion of Wnt proteins. This has served as the basis for the development of Wnt-porcupine inhibitors as WNT974, IWP-2, ETC-159 etc. (Lazzaroni et al., 2016; Kalantary-Charvadeh et al., 2020). Interestingly many inhibitors of this family such as Novartis LGK974 are tested in clinical trials for patients with advanced metastatic solid cancers (Novartis Pharmaceuticals, 2020). Another level of the pathway inhibition is the use of disruptor of the β-catenin/TCF/LEF complexes such as PNU-74654 and PKF118-310 and XAV939. The use of PNU-74654 in association with Ara-C or Idarubicin, abrogate bone marrow protection of AML cells. Similarly, XAV939 suppress T-ALL cell resistance to cytarabine (Leow et al., 2010; Yang et al., 2013; Takam Kamga et al., 2020).

**TABLE 2:** Notch and Wnt inhibitors.

Inhibitors	Cellular target	References
Secreted Frizzled proteins (sFRPs): sFRP1-5	Ligands (Wnt proteins)	Toni et al., 2006
Dickkopf (DKK) proteins: DKK1-4	Receptors (Frizzled)	Chim et al., 2007
Niclosamide	Co-receptors (LRP5/6)	Lu et al., 2011; Takam Kamga et al., 2020
Quercetin	Wnt antagonist promoters/Co-receptors (LRP5/6)	Maso et al., 2014; Alvarez et al., 2018
WNT974	Porcupine	Lazzaroni et al., 2016
IWP-2	Porcupine	Kalantary-Charvadeh et al., 2020; Takam Kamga et al., 2020
ETC-159	Porcupine	Kalantary-Charvadeh et al., 2020
PKF118-310	β-catenin/TCF/LEF	Leow et al., 2010
PNU-74654	β-catenin/TCF/LEF	Takam Kamga et al., 2020
ICAT	β-catenin/TCF/LEF APC-Axin interaction	Pongracz et al., 2006
XAV939	Tankyrase	Yang et al., 2013
Anti-Notch1-4, Anti-Jagged1/2	Receptors	Kamdje et al., 2011; Kamdje et al., 2012
Anti-Jagged1/2, Anti-DLL-1/3-4	Ligands	Kamdje et al., 2011; Kamdje et al., 2012
Gamma secretase-I (GSI-I)	Gamma secretase	Baratta, 2019
GSI-IX (DAPT)	Gamma secretase	Grieselhuber et al., 2013; Takam Kamga et al., 2019b
GSI-XII	Gamma secretase	Takam Kamga et al., 2019a
Others GSI-Is	Gamma secretase	Ran et al., 2017; Baratta, 2019
SHAM1	MALM/RBP-jK	Moellering et al., 2009

Concerning Notch cascade, ligands, and receptors could be targeted by using Notch receptors/Ligand blocking proteins (Kamdje et al., 2011; Kamdje et al., 2012). Several Notch blocking antibodies are used in clinical trials including OMP-52M51 (anti-Notch1), OMP-21M18 (anti-DLL4), OMP-59R5 (anti Notch2/Notch3) (Andersson and Lendahl, 2014; OncoMed Pharmaceuticals, Inc, 2020). Decoy receptors were also developed to interfere with ligand receptors binding (Funahashi et al., 2008). However, the family of gamma secretase inhibitors (GSIs) has been the main source of the development of Notch inhibitors. They present the unique characteristics to inhibits the activity of all receptors. It is indeed an advantage to exclude redundant activity, but it becomes an inconvenient when only one or two receptors are involved in the cancer process (Ran et al., 2017; Baratta, 2019). Ultimately a transcriptional inhibitor of Notch signaling was synthesized, SAHM1. We provided evidence that SAHM1 could interfere with MSC-induced Notch signaling in AML, abrogating drug resistance (Takam Kamga et al., 2016a).

## Conclusion

Stromal microenvironment is the major regulator of drug resistance in leukemia, therefore many studies have tried to dissect the molecular mechanisms supporting the pro-survival role of BMME (Agarwal and Bhatia, 2015). The crosstalk between Notch and Wnt signaling acts as a conserved mechanism to promote the BMME-induced chemoresistance of leukemia cells, regardless the leukemia subtype (Sengupta et al., 2007). These pathways can be targeted at different levels of their cascade through several inhibitors, some of them already used in clinical trials, with different results in terms of outcome and toxicity. Thus, Wnt and Notch inhibitors represent potential therapeutic strategies to target leukemia BMME, regardless the underlying molecular signature, thus minimizing the risk of leukemia subclone selection due to the use of inhibitors of specific molecular aberrations (Rizzo et al., 2008; Ntziachristos et al., 2014; Takebe et al., 2015). Most data supporting this view emerge from co-culture studies between leukemia cells and MSCs. Indeed, MSC-based 2D co-culture cannot address cellular heterogeneity and mechanical constrain observed in a 3D BM (Marino et al., 2019). Nevertheless, all the results were successfully translated into different mouse models, thus confirming that *ex vivo* MSC-leukemia cell coculture can be an effective surrogate to investigate BMME interactions *in vitro* and to pave the way toward the identification of new therapeutical approaches capable of overcoming chemoresistance.

## Author Contributions

PTK designed and wrote the manuscript. RB, GDC, IT, AR, and CT edited the manuscript. AC wrote and edited the manuscript. MK wrote, edited and validated the final version of the manuscript. All authors contributed to the article and approved the submitted version.

## Conflict of Interest

The authors declare that the research was conducted in the absence of any commercial or financial relationships that could be construed as a potential conflict of interest.
